# First person – Julie Fleitz and Clémence Furic

**DOI:** 10.1242/bio.062856

**Published:** 2026-09-28

## Abstract

First Person is a series of interviews with the first authors of a selection of papers published in Biology Open, helping researchers promote themselves alongside their papers. Julie Fleitz and Clémence Furic are co-first authors on ‘Impacts of early-life thermal challenge on development, survival, and physiology in wild great tits’, published in BiO. Julie is a postdoc in the lab of Sophie Reichert at the University of Turku, Finland, investigating how environmental stressors affect species' fitness and adaptation. Clémence is a PhD student in the lab of Sophie Reichert at the University of Turku, investigating the effects of anthropogenic factors on physiology and behaviour of birds.

**Figure:**
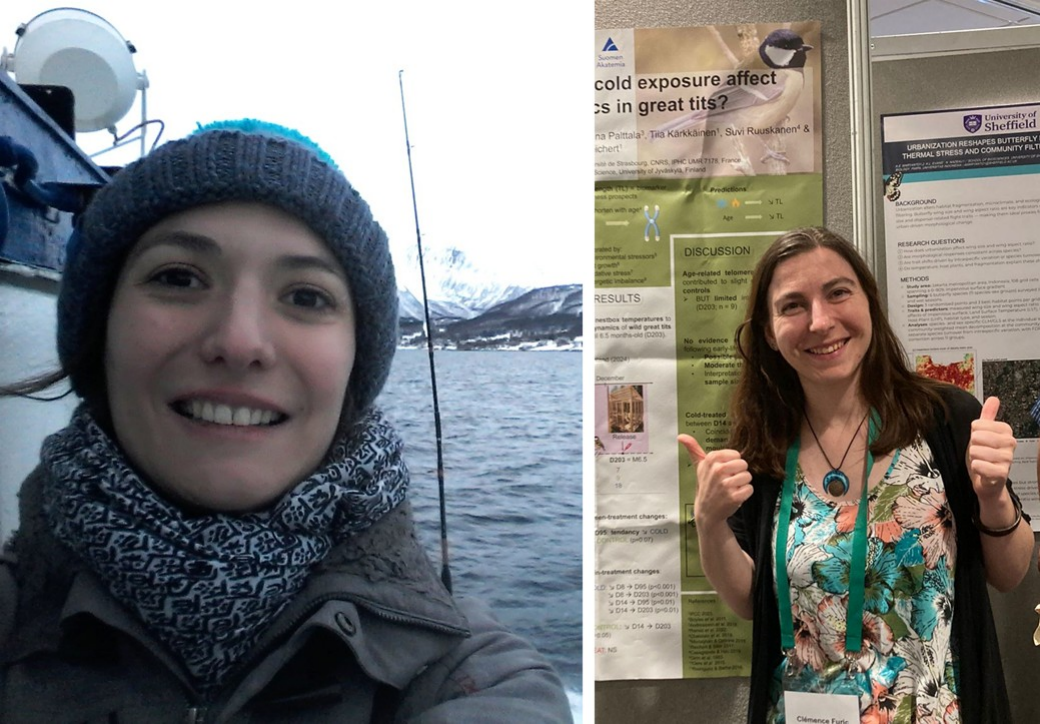
Julie Fleitz (left) and Clémence Furic (right).

**Describe your scientific journey and your current research focus**

Our respective backgrounds are conservation biology (Julie) and ecophysiology/ethology (Clémence). Our current research focuses on the early-life effects of thermal challenges and urbanization on life-history traits, physiology and behaviour of birds. Our projects involve experiments in captive settings and work *in natura*.

**Who or what inspired you to become a scientist?**

**C.F.:** I had the opportunity to watch a few documentaries on biomimicry and was fascinated by how inspiring nature can be and by the countless mysteries it still holds.

**J.F.:** I have always been fascinated by nature and the diversity of species it encompasses. Like Clémence, I've also been captivated by documentaries and by the work of researchers seeking to understand how organisms behave and adapt to a complex, changing world. My decision to pursue a career in science was motivated above all by my passion for biology and by the creative aspect inherent in all scientific research.

**How would you explain the main finding of your paper?**

In our paper, we investigated the immediate effects of a moderate increase and decrease in temperature (around 2°C as climate experts predict for the coming years) during a sensitive stage of development in great tit nestlings, when their ability to regulate their own body temperature is not yet fully developed (between 2 and 8 days old). The experiment was conducted under natural conditions in a Finnish population (Jyväskylä). We found no significant effects of this thermal change on nestling growth, survival, and on some physiological traits, including dehydration or the efficiency of the immune system.

**What are the potential implications of this finding for your field of research?**

Our study suggests that young birds can cope well with small changes in temperature during the first days of their lives. This may be because they are naturally able to handle these conditions, or because their parents help protect them from temperature changes by spending more or less time inside the nestbox. These findings are encouraging, but they do not mean that climate change is harmless. More intense or longer-lasting heat waves could still have negative effects, and some impacts may only appear later in life. Furthermore, other bird populations, such as those found at lower latitudes, may be more sensitive to thermal stress than Finnish populations, which are acclimatised to greater temperature fluctuations throughout the year. Our results highlight the importance of studying how animals of various populations respond to increasingly extreme weather over the long term.

**Figure:**
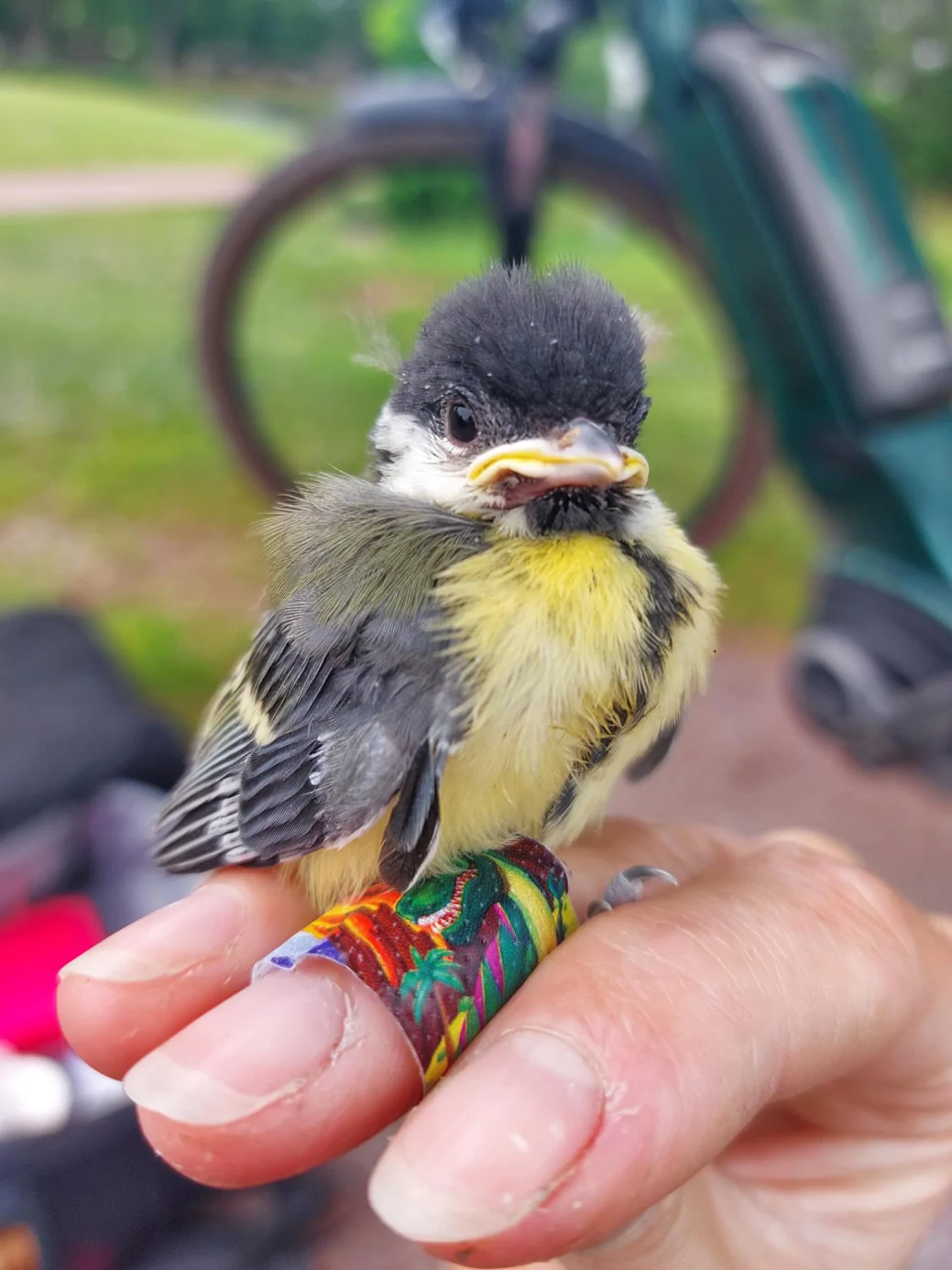
Great tit nestling of 14 days old, waiting to be measured before being placed back into its nest.

**Which part of this research project was the most rewarding?**

This research project has been an enriching experience, as it is the result of a great collaboration between two Finnish research teams (from Turku and Jyväskylä) and other international partners. Furthermore, conducting the experiment in the wild required meticulous organization and unwavering commitment from all parties involved (nearly 500 nest boxes to monitor regularly and the handling of more than 400 nestlings). This teamwork enabled us to collect the consequent dataset presented in this study.

**What do you enjoy most about being an early-career researcher?**

As early career researchers, we enjoy the opportunity to further develop our knowledge on many different aspects, from designing and refining experiments to discovering new laboratory techniques. We also appreciate being involved in every stage of the research process, from developing the experiment to writing the manuscript. Finally, learning about ongoing research projects at conferences and seminars is another highly stimulating aspect of this career.

**What piece of advice would you give to the next generation of researchers?**

We would encourage the next generation to always remain curious and open to learning new techniques and approaches. Researchers are generally very willing to share their knowledge, whether they are members of your own team or people you meet at conferences. Science can be a challenging career, with many ups and downs, and we would advise young researchers to be bold in reaching out to everyone working in a scientific lab and to seek help whenever needed. Whether it is about the best way to conduct an experiment, analyse data, or explore career opportunities, do not hesitate to ask questions, you never know what might come your way.

**What's next for you?**

**J.F.:** My postdoc will end next year, and I am currently applying for several international grants to start developing more independent research projects. These projects aim to combine the expertise developed during my PhD, which mainly focused on conservation biology, with the techniques and knowledge I have gained during my current postdoc, particularly in the field of ecophysiology.

**C.F.:** I will finish my PhD next year, and I'm exploring what opportunities are available, mostly related to my PhD, but some focusing on a different bird model. I would also love to build new collaborations with both my current collaborators and new research teams.
